# An Update on the Nutritional and Therapeutic Potential of *Dioscorea oppositifolia*

**DOI:** 10.1002/fsn3.70179

**Published:** 2025-05-05

**Authors:** Ruchika Kumari, Ankita Thakur, Palak Thakur, Vipasha Sharma, Rohit Sharma, Sachin Upmanyu, Randeep Singh, Zainab M. Almarhoon, Daniela Calina, Javad Sharifi‐Rad, Ashun Chaudhary

**Affiliations:** ^1^ Department of Plant Sciences, School of Life Sciences Central University of Himachal Pradesh Dharamshala India; ^2^ Department of Forest Products, College of Forestry Dr. Yashwant Singh Parmar University of Horticulture and Forestry Solan India; ^3^ PG Department of Zoology Khalsa College Amritsar Punjab India; ^4^ Department of Chemistry, College of Science King Saud University Riyadh Saudi Arabia; ^5^ Department of Clinical Pharmacy University of Medicine and Pharmacy of Craiova Craiova Romania; ^6^ Universidad Espíritu Santo Samborondón Ecuador; ^7^ Centro de Estudios Tecnológicos y Universitarios del Golfo Veracruz Mexico; ^8^ Department of Medicine, College of Medicine Korea University Seoul Republic of Korea

**Keywords:** anticancer, antidiabetic, anti‐inflammatory, antimicrobial, antioxidant, *Dioscorea oppositifolia*, phytochemicals, therapeutic potential

## Abstract

*Dioscorea oppositifolia* L., also known as Cinnamon vine, has a longstanding history in traditional medicine due to its diverse bioactive compounds. However, comprehensive scientific evaluations of its phytochemical properties and therapeutic potential are still limited. This study provides an updated analysis of its nutritional composition, bioactive compounds, and pharmacological properties, emphasizing potential therapeutic applications. A comprehensive literature review was conducted using Google Scholar, Science Direct, and PubMed/MedLine, covering studies published between 2022 and 2023. Key bioactive constituents such as diosgenin and dioscorine were identified and evaluated for their biological activities, including antioxidant, anti‐inflammatory, antidiabetic, anticancer, and antimicrobial effects. The plant exhibits notable antioxidant activity by reducing oxidative stress, while its anti‐inflammatory effects are linked to cytokine inhibition. Antidiabetic properties are evidenced by blood glucose modulation and improved insulin sensitivity. Anticancer effects include apoptosis induction and suppression of cancer cell proliferation. Additionally, *Dioscorea oppositifolia* shows antimicrobial efficacy against various bacterial and fungal strains. Due to its broad‐spectrum bioactivities and rich phytochemical profile, *Dioscorea oppositifolia* emerges as a promising candidate for pharmaceutical applications. However, further research, including clinical trials and bioavailability studies, is essential to fully harness its therapeutic potential and facilitate its integration into modern medicine.

## Graphical abstract

*Dioscorea oppositifolia* L. (Cinnamon vine) contains bioactive compounds, such as diosgenin, demonstrating anticancer properties. Diosgenin inhibits JAK/STAT3 signaling, suppressing cell proliferation, invasion and metastasis. Additionally, it triggers mitochondrial apoptosis by activating Bax, promoting cytochrome C release and stimulating caspase‐9 and caspase‐3 activation, ultimately leading to cancer cell apoptosis.

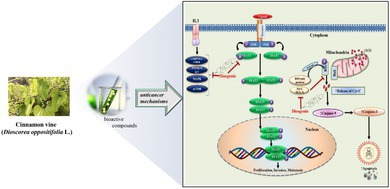

**Abbreviations**
ABTS: 2,2′‐azino‐bis(3‐ethylbenzothiazoline‐6‐sulfonic acid)
AMPK: AMP‐activated protein kinase
ATGL: adipose triglyceride lipase
Bcl‐xL: B‐cell lymphoma‐extra large
CML: chronic myeloid leukemia
COX‐2: cyclooxygenase‐2
COXs: cyclooxygenases
CPT: carnitine palmitoyl transferase
DOAJ: Directory of Open Access Journals
DPPH: 2,2‐diphenyl‐1‐picrylhydrazyl
Erβ: estrogen receptor beta
FAS: fatty acid synthase
FoxO1: forkhead box O1
gL: gram/l
HIV: human immunodeficiency virus
IL‐6: interleukin 6
LDL: low‐density lipoprotein‐cholesterol
LOXs: lipoxygenases
LXRα: liver X receptor α
Mcl‐1: myeloid cell leukemia 1
MMP‐2: matrix metalloproteinase‐2
NAFLD: non‐alcoholic fatty liver disease
PPARγ: peroxisome proliferator‐activated receptor gamma
ROS: reactive oxygen species
SCD: stearoyl‐CoA desaturase
SIRT1: silent information regulator of transcription factor 1
SREBP‐1c: sterol regulatory element‐binding protein 1c
STAT3: signal transducer and activator of transcription 3
STZ: streptozotocin
TG: triglycerides
TNF‐α: tumor necrosis factor alpha

## 1 Introduction

Nature provides a wide range of wild edible medicines, which have sparked renewed interest in recent years (Jiang et al. 2023). Plants have been present on earth since the beginning of life and are the foundation of traditional medicines worldwide (Thakur et al. 2024). WHO estimates that 80% of people use traditional medicines for essential medical care and large populations in rural areas use these medicines to treat many ailments because of their low cost and ease of access (Chaudhary et al. 2023). Tubers and root crops are pre‐eminent food crops following cereals. Tubers that develop naturally without cultivation or attention are called wild tubers. These plants primarily flourish in forests, untamed areas, farming margins, and arid fields. People have consumed wild tubers for thousands of years, but regrettably, we lost sight of this practice at the beginning of the modern era. However, these wild tubers are still valuable to rural and tribal communities. Tuber crops not only provide nutritional requirements to humans but also possess therapeutic characteristics that can treat various illnesses (Edison et al. 2006). The genera *Dioscorea* has been regarded as one of the earliest angiosperms, originating in the Indo‐Malyan and Southeast Asian region with more than 600 species. Only seven species out of 600 species are commonly consumed in West Africa, nine are present in China, while in Taiwan 14 species are present (Padhan and Panda 2020). There have been reports of around 50 distinct species of *Dioscorea* in India, which have been reported from states like Tamil Nadu, Assam, Bihar, West Bengal, Kerala, Rajasthan, Odisha, and Gujarat (Kumar et al. 2017). *D. oppositifolia* , *D. pentaphylla* , *D. bulbifera* , etc. are the most common species of *Dioscorea*. Among all of the species of *Dioscorea*, the most economically substantial species is *D. alata* , which is native to Southeast Asia, especially to Thailand and Tropical Myanmar. *D. bulbifera* is another well‐known species that has been studied, having a vast native range that extends over most of Northern Australia, Asia, and Tropical Africa (Kumar et al. 2017) (Narayan et al. 2020). Due to the availability of essential nutritional components, *Dioscorea* has been a significant source of diet for many indigenous people and is a staple meal for poor people in various areas. Water is the main constituent of *Dioscorea*; for example, in *D. pentaphyla*, *D. bulbifera*, and *D. delicata*, 93% of the constituent is water in fresh weight of the tuber, whereas the other species of *Dioscorea* moisture content ranges from 51% to 90% (Mohan and Kalidass 2010) (Shanthakumari et al. 2008). The *Dioscorea* species contain high quantities of carbohydrates, starch, fiber, and sugar, making it a possible dietary source. The sufficient intake of fiber enhances regular bowel movements, reduces intestinal transit, fecal bulkiness and boosts the body's ability to hold onto water. *Dioscorea oppositifolia* is one of the important wild tubers that belong to the family *Dioscorea*ceae and is referred to as a cinnamon vine or cinnamon yam (Paul et al. 2014). It is a climber plant with a single perennial tuber (root‐stock), and a light purplish stem (Kumar et al. 2012), that is cylindrical, and descends far into the ground. On the eastern and western ghats, this tuber is referred to as the “most‐favored” tuber by the locals (Edison et al. 2006). Although bulbils are occasionally harvested and used as food, *D. oppositifolia* tubers and bulbils can both be eaten. *D. oppositifolia* consist of essential nutritional components which stimulate positive physiological effects like lowering sugar levels in the blood along with cholesterol levels, eradicating toxins, as well as *promoting* the development of the gut's normal microbial flora. Proteins, fats, vitamins, and minerals are among the additional nutritional elements present in this species (Obidiegwu et al. 2020). *Dioscorea* also produces bioactive plant compounds, which provide pharmacological or toxicological effects to these wild tubers. A bioactive molecule is a substance that possesses biological activity and can elicit a response or reaction in living tissue (Guaadaoui et al. 2014). Studies have demonstrated that the biologically active compounds present in *D. oppositifolia* have medicinal potential. Numerous Secondary metabolites have been identified in several varieties of *Dioscorea*, including polyphenols, saponin, phenolics, alkaloids as well as flavonoids, dioscorin, allantoin, dioscin, and diosgenin (Siadjeu et al. 2020; Wang et al. 2023). The majority of species possess steroid sapogenins and saponins that include diosgenin, which serves as the building block to synthesize several steroidal hormones utilized as androgenic, anti‐inflammatory, estrogenic as well as contraceptive medications (Dutta 2015). *D. oppositifolia* has an extensive variety of bioactivities, including antioxidant, anti‐inflammatory, anticancer, and antibacterial properties, which can be directly correlated with this broad spectrum of phytochemicals (Rani and Raju 2020). Diosgenin is present in its root, which is often used in the manufacturing of progesterone and various steroidal drugs. Traditionally, it has been utilized as a contraception and to treat a variety of genital disorders (GISD 2023). *D. oppositifolia* exhibits saponin, terpenoid, and cardiac glycosides that have the potential to treat bacterial infections, fungal and yeast infections, as well as cardiac failure and are used traditionally for the treatment of digestive ailments (Jeong et al. 2016; Sheikh et al. 2013). In addition to being used as an herbal tonic, the tubers of this plant are eaten to cure depression, low appetite, diabetes, dry coughs, asthma, recurrent or uncontrollable urination, and chronic diarrhea. The tubers are externally utilized to treat ulcers, abscesses, and boils, as they possess allantoin, which promotes cell growth and accelerates the process of healing. The leaf juice of this plant is used to cure scorpion stings and snake bites. Thus, an attempt has been made to investigate the ethnobotany, secondary metabolites, and other pharmacological investigations of *D. oppositifolia*. Pharmacological research has led to the discovery of most plant phytochemicals used in modern health care. Although its therapeutic potential has not been extensively studied so far, its traditional knowledge‐based systems have shown great use for various biological purposes. The purpose of this study is to provide a comprehensive evaluation of the known secondary metabolites of this plant and its diverse applications, as well as to provide new avenues of investigation for those working in the fields of phytomedicine and clinical research.

## 2 Review Methodology

**FIGURE 1:**
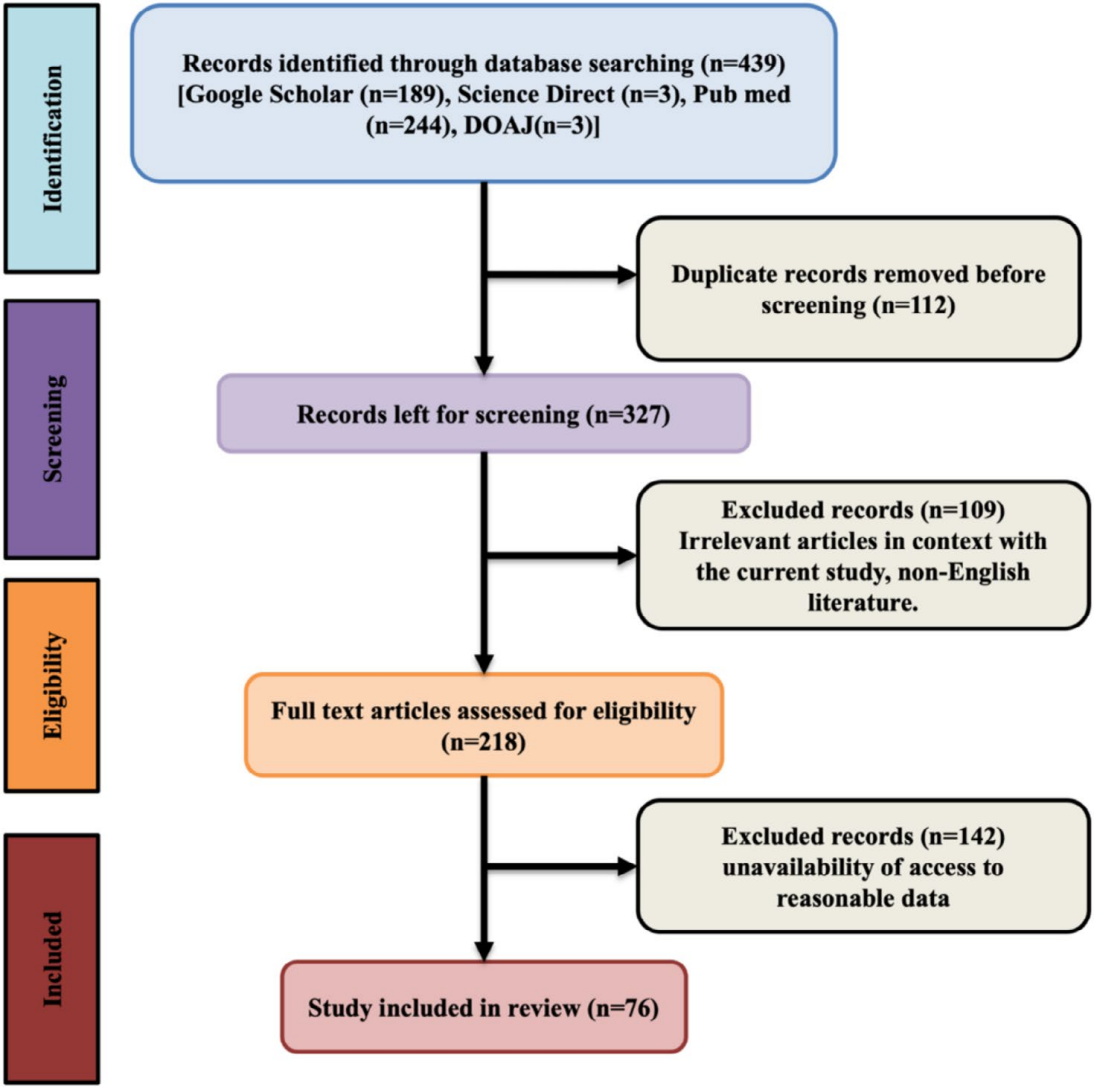
Flowchart of the literature review process for research article selection. Initially, 439 records were identified across several databases. Duplicates were removed, and 327 records were screened. Post‐screening, 218 articles were assessed in full text, with 142 excluded due to various reasons, resulting in 76 studies being included in the final review.

To gather information on the botanical description, distribution, medicinal usage, ethnobotanical use, bioactive compounds, nutritional aspects, and pharmacology of *D. oppositifolia* , extensive online literature available on various portals was investigated from 2022 to 2023. Keywords used included “ *D. oppositifolia* ,” “ethnobotany,” “bioactive compounds,” “nutritional composition,” and “pharmacology.” The preferred language for this study was English. The complete methodology is divided into three main parts: identification, screening, and research (Figure 1) (Lo 2022). Publications were identified through networks such as Google Scholar, ScienceDirect, and PubMed/MedLine, among others. Before screening, a reference manager detected and eliminated duplicate articles. During the initial screening, non‐English literature, irrelevant articles, and full texts and abstracts that could not be retrieved were removed. Studies not pertinent to the review were also eliminated. Reported phytochemicals were checked using the PubMed database, and ChemDraw was used to draw chemical structures. The study focused on the main objectives, including nutritional aspects, ethnobotanical, and pharmacological research.

## 3 Botanical and Ethnobotanical Profile of *Dioscorea oppositifolia*

### 3.1 Distribution and Habitat

India has a wide range of tuber and root crops, where the Western Ghats and North‐eastern region are the world's biodiversity hotspots. The distribution of tuber and root crops in India is divided into five major regions: the southern peninsular region, the south‐western hilly and coastal area, the eastern coastal area, the north‐western zone, and the north‐eastern zone. *D. oppositifolia* is found in moist deciduous and interior evergreen forests of India, Bangladesh, the East Himalayas, Myanmar, and Sri Lanka (Edison et al. 2006). In India, it is distributed in Odisha, Kerala, Arunachal Pradesh, Andhra Pradesh, Odisha, Assam, Karnataka, and Tripura (Paul et al. 2014).

### 3.2 Botanical Description

*D. oppositifolia* is a twining vine that grows quickly and spreads out of control but has evaded cultivation. Although it may thrive in a variety of habitats and environments, this species is usually found on the edges of lush, mesic bottomland forests, near streams, drainage channels, and fence rows. This evergreen, twining vine belongs to the family of yam *Dioscoreaceae* (Coursey 1967). It is a deciduous twining vine with 3–4 m of height that creeps throughout the year with sparsely hairy tubers (Lim 2016). Although the leaves are often placed opposite to one another and alternate at the higher nodes, they also follow ternate phyllotaxy, which means three leaves are attached at each node (Coursey 1967). It has simple leaves, including an ovate, hastate, or sagittate shape that is 4–8 cm long and 7–9 nerved (veined). The leaf margins, petioles, and stems are often reddish‐purple, and the bases of the leaves are strongly lobed, while the tip is acuminate. New leaves frequently have a distinctive bronze hue. Flowers are tiny, greenish‐yellow (white), and scented with cinnamon. The plants are dioecious, while the flowers are unisexual and appear as spike‐ or paniculate‐shaped inflorescences in the leaf axils. Fruits are membranous with triangular capsules (GISD 2023).

### 3.3 Ethnobotanical Uses

Ethnobotany is a multidisciplinary field primarily concerned with how humans in particular cultures and regions utilize plants. Throughout history, humans have depended on the natural world to fulfill their fundamental needs, finding fascination and utility in the variety of plant life around them (Rahman et al. 2019). Most of the plant secondary metabolites that have been utilized in modern medicine are reported via ethnobotanical research. Several species of *Dioscorea* retain a prominent role in traditional medication to address a variety of disorders, as the root syrup of *Dioscorea* species is utilized to ease labour pain, and physicians also suggest it to people who suffer from colic discomfort, rheumatism, asthma, and stomach issues that are associated with alcoholism (Padhan and Panda 2020). Powder of tubers has been included in cholera and constipation medications, and juice of the plant has been applied to wounds, to cure piles, intestinal worms, skin‐related diseases, and obesity (Chandrasekara and Josheph Kumar 2016). *D. oppositifolia* is well known for its therapeutic, culinary, and commercial benefits. Traditionally, *D. oppositifolia* is used for abscesses, ulcers, antiseptics, and roots are chewed to treat toothache and aphthae. Secondary syphilis and psoriasis are treated with whole plant extract (Felix et al. 2009). It has been utilized to stimulate the stomach and spleen. A tuber of *D. oppositifolia* is occasionally used in herbal tonics and has been utilized to treat anxiety, persistent diarrhea, dry coughs, asthma, frequent or involuntary urination, and reduced appetite (Poornima and Ravishankar 2007). The *Dioscorea* species tubers are boiled to eradicate the bitterness, and curry is made as food for direct consumption (Mallick et al. 2020). The *Dioscorea* species contain a high amount of carbohydrates, fats, starch, and sugar, making it a possible dietary source. Nutritional analysis of *D. oppositifolia* reveals that the tuber possesses 80% water, 20% starch, and 0.1% sugar (GISD 2023). Numerous Secondary metabolites have been reported in several varieties of *Dioscorea* that include polyphenols, saponin, phenolics, alkaloids, as well as flavonoids, dioscorin, allantoin, dioscin, diosgenin (Wang et al. 2023). Diosgenin is in higher demand in the market value and the breakdown of diosgenin derivatives results in the yield of diosgenin, which is the key source of anti‐inflammatory, corticosteroids, androgen, estrogen, and contraceptives medications (Nazir et al. 2021; Wang et al. 2023). *D. oppositifolia* exhibits a wider range of bioactivities that include antioxidant, anti‐inflammatory, anticancer, and antibacterial properties, in addition to a broad spectrum of phytochemicals (Rani and Raju 2020). *Dioscorea* has various ethnobotanical uses and their methods of preparation, which are listed in Table 1.

**TABLE 1:** Ethnobotanical uses of *D. oppositifolia* .

Botanical name	Plant part	Ethnobotanical uses	References
*D. oppositifolia*	Tuber	Tubers are given to women, which have been boiled with *D. uniflorus* , once daily for almost a month after delivery to regain their strength.	(Gavad and Khade 2021)
		For sperm production, honey and tuber powder are administered orally.	(Kumar et al. 2017)
	Leaf	To cure ulcers, leaf paste is used as an antiseptic.	(Kumar et al. 2017)
	Root	On a scorpion bite, root powder with cow urine is applied.	(Kumar et al. 2017)
	Tuber	Utilized to cure epilepsy.	(Kumar et al. 2017)
	Leaves, flowers, tender shoots, and tubers	Used for cooling and demulcent. *D. oppositifolia* leaves are blended with clematis leaves, and 2–3 drops of liquid are put in the affected person's nose.	(Felix et al. 2009)
	Leaves, flowers, tender shoots, and tubers	Utilized as a decoction for cancerous lesions and leprosy.	(Felix et al. 2009)
	Root	Chewed to treat aphtha and toothache.	(Felix et al. 2009)
	Flowering twigs	Leprosy is cured by ash extract from flowering twigs and tender leaves.	(Felix et al. 2009)
	Whole plant	The whole plant extract is used to treat psoriasis and secondary syphilis.	(Felix et al. 2009)
	Rhizome	Rhizome juice is consumed to minimize menopause‐related problems.	(Dash et al. 2022)
	Tuber	For early menstruation, a tuber decoction is utilized.	(Dash et al. 2022)
		The tubers decoction is utilized as a contraceptive and to fight obesity.	
		To stimulate appetite, the tuber is consumed after being boiled.	

## 4 Nutritional Benefits

**FIGURE 2:**
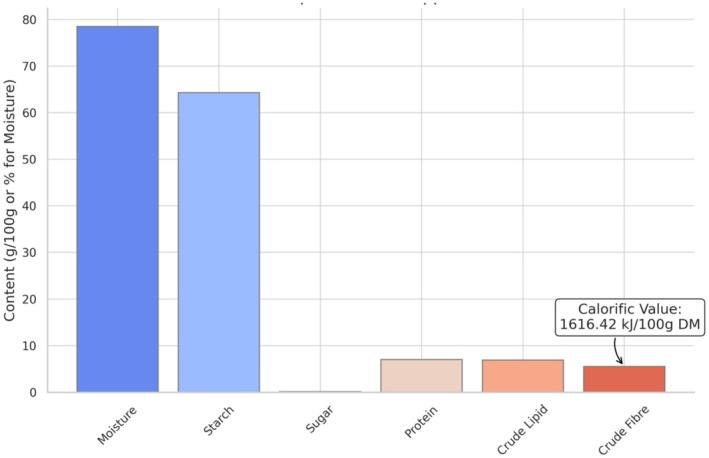
Nutritional composition of *D. oppositifolia* tubers.

In terms of nutrition, tubers and roots have a lot of potential in the form of carbohydrates to offer affordable sources of energy (Figure 2 and Table 2) (Chandrasekara and Josheph Kumar 2016). The tubers appeared to be a reliable source of various dietary elements and contain a substantial fraction of protein, minerals, and essential amino acids (Darkwa et al. 2020). *D. oppositifolia* tubers and bulbils are both consumable, while the bulbils of this plant are rarely harvested and consumed as food. The tuber possesses 80% water, 20% starch, and 0.1% sugar. It also comprises glutamine, mucilage, amylase, 10 to 15 mg of vitamin C, and vitamin B1 (GISD 2023). Both the nutritional content and the analytical techniques for the quantification of *Dioscorea* are highly significant. Moisture content is the primary constituent in *D. oppositifolia* , which contributes up to 78.49% moisture. The amount of moisture in tubers and roots is important for extending product shelf life and also identifying how susceptible a crop is to microbial breakdown.

**TABLE 2:** Comprehensive nutritional profile of *D. oppositifolia* .

Nutritional component	Content in *D. oppositifolia*	Importance for human health	References
Water content	80%	High water content suggests significant implications for storage and microbial resistance.	(GISD 2023)
Starch	20%	Starch as the primary carbohydrate source underscores its role as an energy staple.	(GISD 2023)
Sugars	0.1%	Low sugar content aligns with health‐conscious dietary trends.	(GISD 2023)
Protein	7.00 ± 0.07 g/100 g	Comparable to other protein sources, highlighting its nutritional value.	(Shanthakumari et al. 2008)
Crude lipid	6.92 ± 0.11 g/100 g	Higher lipid content than other *Dioscorea* species, indicating a richer energy source.	(Shajeela et al. 2011)
Crude fiber	5.53 ± 0.13 g/100 g	Contributes to dietary fiber intake, beneficial for digestive health.	(Shanthakumari et al. 2008)
Vitamin C (ascorbic Acid)	10 to 15 mg/100 g	Essential for immune function; the range indicates variability possibly due to environmental factors.	(GISD 2023)
Vitamin B1	Present	Supports energy metabolism, enhancing the tuber's dietary value.	(GISD 2023)
Glutamic acid	8.12 g/100 g	High levels suggest a robust profile for protein synthesis and metabolic functions.	(Doss et al. 2019)
Aspartic acid	9.36 g/100 g	Its abundance aligns with the strong amino acid profile of *D. oppositifolia* .	(Doss et al. 2019)
Niacin	5.49 ± 0.48 mg/100 g	Vital for energy release from food, reflecting the tuber's nutritional efficiency.	(Shanthakumari et al. 2008)
Potassium	1460.41 ± 0.13 mg/100 g	High potassium levels are key for cardiovascular health and electrolyte balance.	(Shanthakumari et al. 2008)
Magnesium	548.33 ± 0.11 mg/100 g	Supports bone health and enzymatic reactions	(Shanthakumari et al. 2008)
Calcium	124.00 ± 0.03 mg/100 g	Essential for bone health and neurological functions.	(Shanthakumari et al. 2008)
Sodium	113.00 ± 0.14 mg/100 g	Moderate sodium content is conducive to maintaining healthy blood pressure levels.	(Shanthakumari et al. 2008)
Calorific value	1616.42 kJ/100 g DM	Indicates the tuber's high energy content, relevant for nutritional planning.	(Shanthakumari et al. 2008)

**FIGURE 3:**
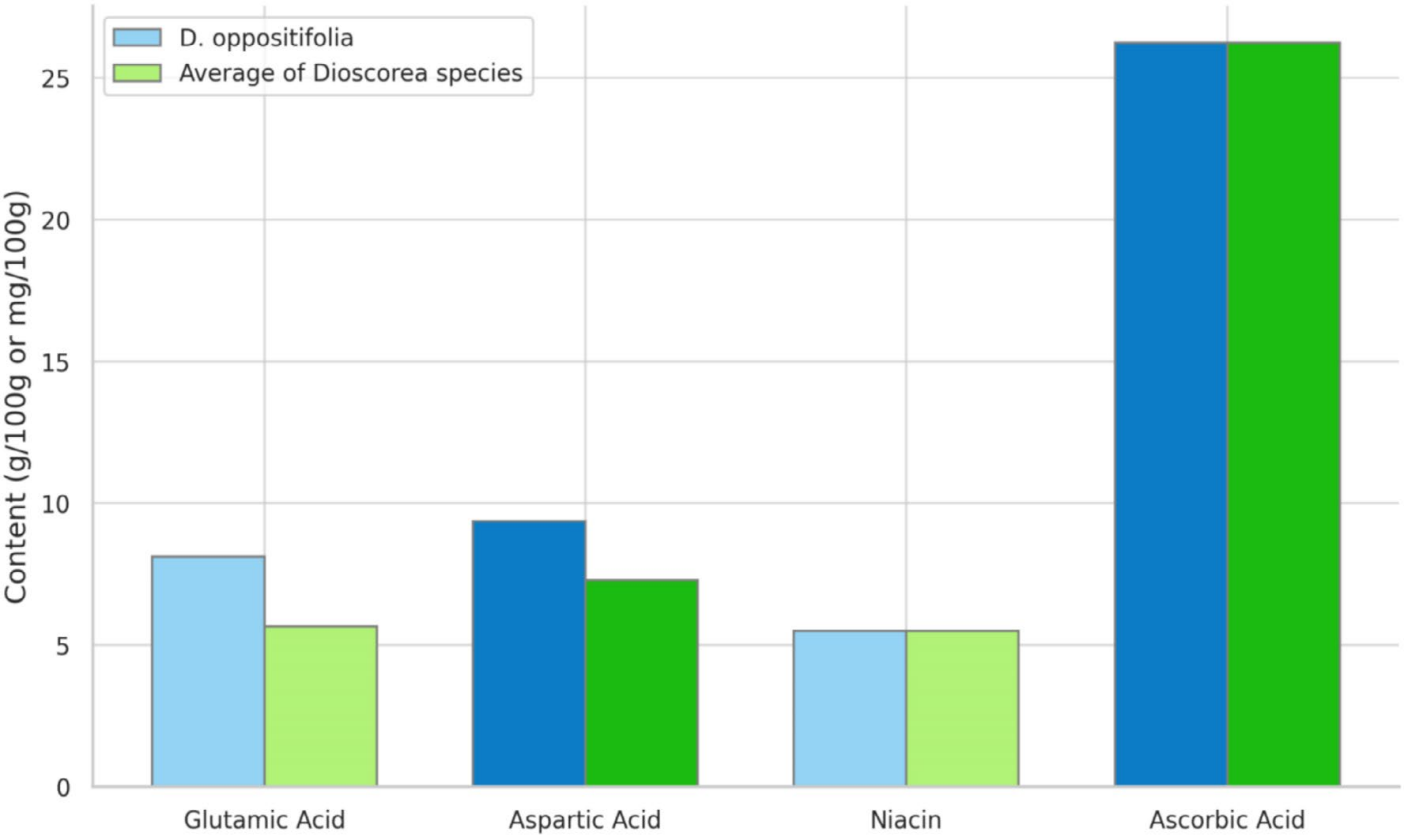
Comparative diagram between the amino acids and vitamins in *D. oppositifolia* with other *Dioscorea* species.

The essential amino acids threonine and phenylalanine are particularly abundant in *Dioscorea* tubers, whereas tryptophan amino acids are limited (Figure 3). According to the amino acid profiling of various *Dioscorea* species, including *D. bulbifera* , *D. alata* , *D. pentaphylla* , *D. esculenta* , *D. spicosa*, *D. oppositifolia* , *D. wallichi*, and *D. tomentosa* , it was revealed that glutamic acid 3.20 to 8.12 g/100 g (glutamic acid), and 5.21 to 9.36 g/100 g (aspartic acid) are prevalent in all the species (Doss et al. 2019). Shajeela et al. reported that in *D. oppositifolia* , the crude lipid content is higher as compared to the other species of *Dioscorea* (Shajeela et al. 2011). The proximate content of crude lipid in *D. oppositifolia* tubers is 6.92 ± 0.11 (g 100 g^−1^), with a moisture content of 78.49%. *D. oppositifolia* tubers were reported to contain 7.00 ± 0.07 (g 100 g^−1^) of crude protein and 5.53 ± 0.13 (g 100 g^−1^) of crude fiber. The calorific value was determined to be 1616.42 (kJ 100 g‐^1^ DM). The contents of starch and vitamins in *D. oppositifolia* are found to be 64.29 ± 0.51 (g/100 g) of starch, 5.49 ± 0.48 (mg 100 g ^−1^) of niacin, and 26.23 ± 0.12 (mg 100 g^−1^) of ascorbic acid (Shanthakumari et al. 2008). According to mineral analysis, *D. oppositifolia* has high potassium levels (1460.41 ± 0.13 mg/100 g), magnesium levels (548.33 ± 0.11 mg/100 g), calcium levels (124.00 ± 0.03 mg/100 g), and sodium levels (113.00 0.14 mg/100 g) (Shanthakumari et al. 2008).

## 5 Phytoconstituents

The term bioactive compounds refers to secondary metabolic products of plants with pharmaceutical or toxicological impact on humans and other animals. In addition to major metabolic and biosynthetic pathways for molecules that contribute to plant development and growth, secondary metabolites are the compounds that are produced within plants (Bernhoft 2010). Plant‐based bioactive compounds greatly aid the prevention of several human diseases, and these bioactive compounds are produced through a complex process that is controlled via environmental circumstances (Semwal et al. 2021).

### 5.1 Saponins

**FIGURE 4:**
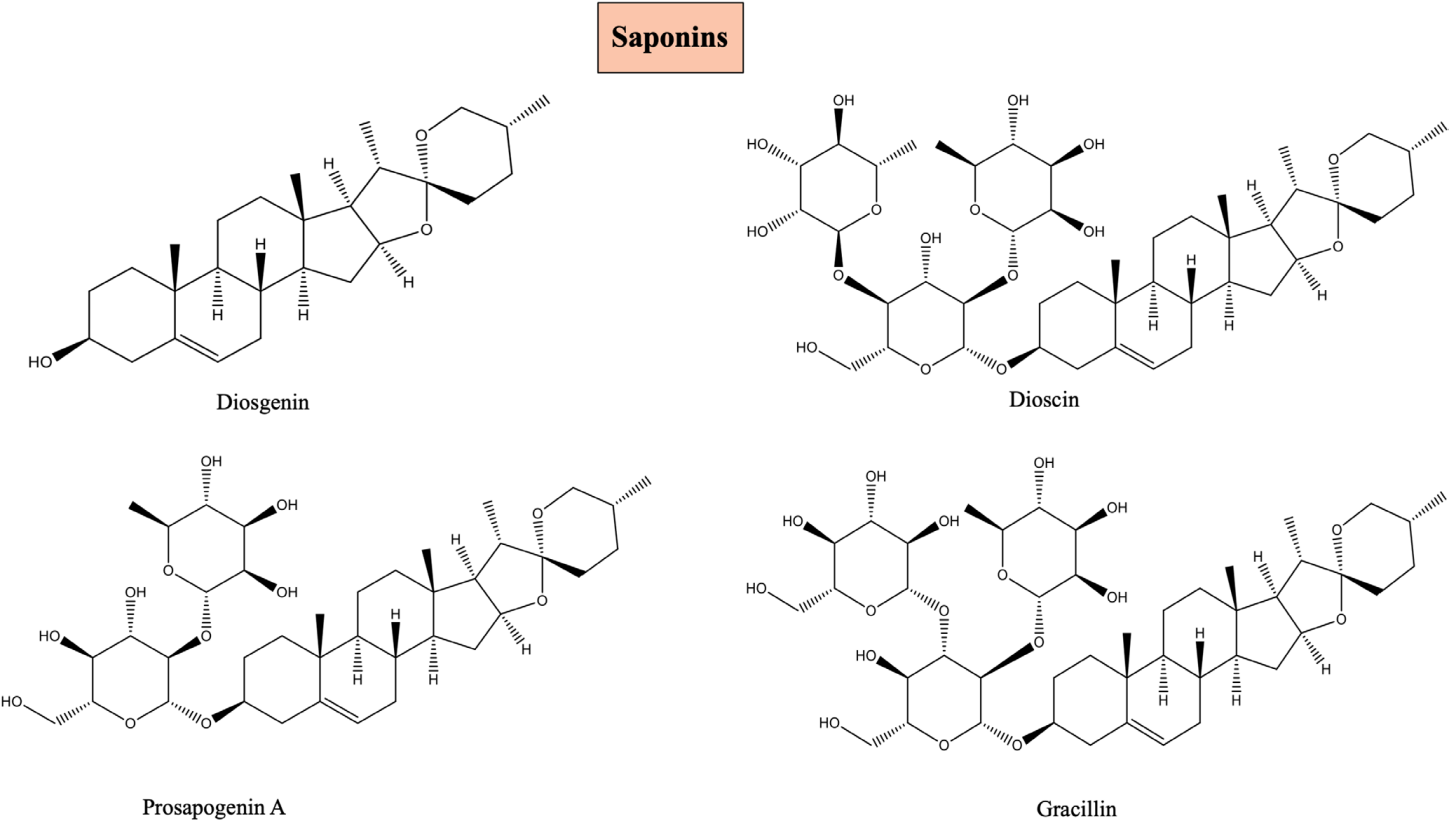
Steroidal saponins identified in Dioscorea oppositifolia, including diosgenin, dioscin, prosapogenin A, gracillin, and trillin, known for their antioxidant and lipid‐regulating effects.

Saponins are a large class of glycosidic chemical substances made up of triterpenoid and steroidal aglycones. Due to the variety of their structural components, including sugars and aglycones, SS (Steroidal saponins) demonstrate an extensive range of biological activity (Escobar‐Sánchez et al. 2015). SS can be divided into stigmastane, spirostane, cholesane, furostane, pregnane, and ergostane families depending on the aglycone moiety (Obidiegwu et al. 2020). The pharmaceutical industry has utilized a well‐known natural SS diosgenin as a chemical basis in the full manufacturing of hormonal medications. This metabolite has been shown to protect the myocardium from ischemia‐induced damage and to prevent oxidative stress damage. Diosgenin, trillin, β‐daucosterol, prosapogenin A, dioscin, and gracillin are the saponins that are found in *D. oppositifolia* (Zhao et al. 2019) (Figure 4). A pharmacologically viable steroidal saponin called trillin, which is present in *D. oppositifolia* , has a variety of physiological effects on cells, including upregulating superoxide dismutase (SOD) activity, downregulating the oxidative stress response, and lipid peroxidation activity (Adomėnienė and Venskutonis 2022).

### 5.2 Alkaloids

**FIGURE 5:**
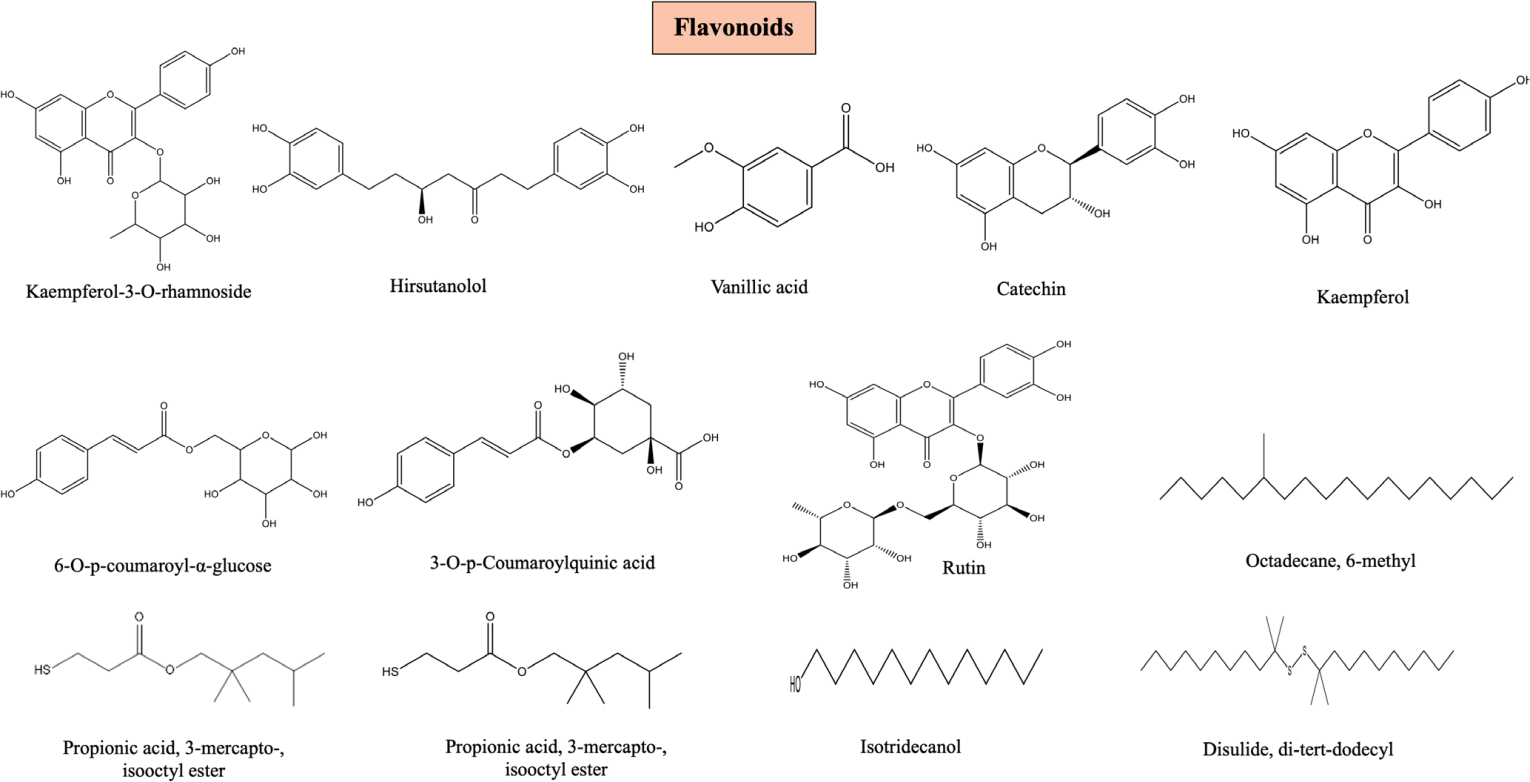
Different flavonoids and alkaloids are present in *D. oppositifolia* .

Alkaloids belong to an immense and structurally diversified class of heterocyclic (Cushnie et al. 2014). Alkaloids have been used in pharmaceuticals due to their medicinal effects, including those against microbes, hypertension, cancer, inflammation, and the human immunodeficiency virus (HIV); a few of these alkaloids are extremely hazardous to both animals and humans. Diosbulbin B, Ethyl iso‐allocholate, Dumetorine, dioscorine, Allantoin, dioscoretine, and dihydrodioscorine are some of the alkaloids that are mainly found in *Dioscorea* tuber (Wang et al. 2023) (Zhao et al. 2019) (Figure 5). *D. oppositifolia* contains alkaloids in varying concentrations ranging from 7.2 to 16 (mg/100 g dry mass) (Obidiegwu et al. 2020). Numerous species of *Dioscorea* (*D. pubera*, *D. hamiltonii*, *D. alata* , *D. oppositifolia* , *D. bulbifera* , *D. wallichi*, *D. pentaphylla* , *D. hispida* and *D. glabra*) have been shown to contain alkaloids with concentrations ranging from 7.2 to 16 mg per 100 g dry weight (Padhan and Panda 2020).

### 5.3 Flavonoids

Flavonoids (Fv), a class of widely dispersed secondary metabolites with various metabolic activities, have different phenolic structures. These organic compounds are well recognized for their positive impact on health efforts, which are being undertaken to extract these components because of their anti‐mutagenic, anti‐inflammatory, antioxidant, and anti‐carcinogenic activities, besides their ability to regulate important cell enzyme processes (Panche et al. 2016). Kaempferol‐3‐O‐rhamnoside, Rutin, Quercetin, Kaempferol, and Catechin are a few flavonoids that are found in *D. oppositifolia* (Zhao et al. 2019) (Figure 5). The flavonoid content of 9 different species of *Dioscorea* that include *D. pubera*, *D. alata* , *D. bulbifera* , *D. oppositifolia* , *D. pentaphylla* , *D. halmitonii*, *D. wallichi*, *D. glabra* , and *D. hispida* revealed that the levels of flavonoid observed in *D. hispida* and *D. alata* are much lower than those in the other *Dioscorea* species, with flavonoid content ranging between 0.62 and 0.85 mg per g dry weight (Padhan and Panda 2020).

### 5.4 Phenolic Acid and Phenols

**FIGURE 6:**
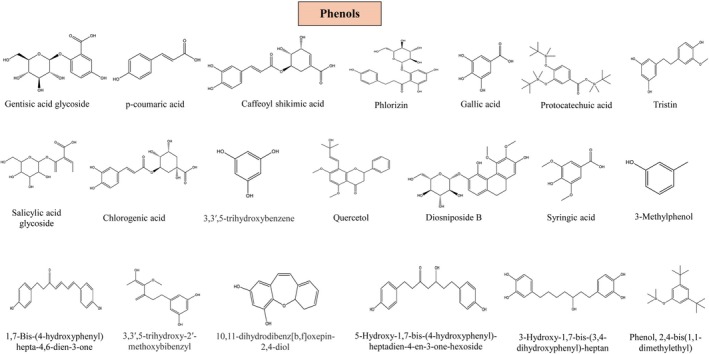
Phenolic compounds present in *D. oppositifolia* .

Phenolic acid and phenols are two of the most prevalent secondary metabolites that have been discovered in plants. *Dioscorea* has been identified to contain phenol and phenolic acid (Obidiegwu et al. 2020) (Figure 6). Numerous phenols and phenolic acids present in *D. oppositifolia* are Gentisic acid glycoside, 1,7‐Bis‐(4‐hydroxyphenyl) hepta‐4,6‐dien‐3‐one, Vanillic acid, Syringic acid, Protocatechuic acid, Gallic acid, Chlorogenic acid, and 3‐Methylphenol. Zhao et al. via using HPLC system assessed the total phenolic content of *D. oppositifolia* and *D. halmitonii* and as a result found that both of the species possess phenolic acid, but the content of phenolic acid in *D. oppositifolia* (297.3 mg per mL) is nearly twice as compared to *D. halmitonii* (158.2 mg/gL), which resulted in significantly improved immunological control, antioxidant, and anti‐inflammatory benefits (Zhao et al. 2019). Additionally, Padhan and Panda (2020) reported a significant difference in the phenolic content ranging between 2.1 and 9.62 mg per g dry weight of several *Dioscorea* species (*D. pubera*, *D. alata* , *D. bulbifera* , *D. oppositifolia* , *D. wallichii* , *D. hamiltonii*, *D. pentaphylla* , *D. glabra*, and *D. hispida* ) (Padhan and Panda 2020). Tables 3 and 4.

**TABLE 3:** Phytoconstituents in *D. oppositifolia* and their biological activities.

Phytoconstituent	Specific compounds in *D. oppositifolia*	Biological activities	Concentration	References
Steroidal saponins	Diosgenin, trillin, β‐daucosterol, prosapogenin A, dioscin, gracillin	Cardioprotective, antioxidative stress	10 mg/kg dry weight in the other species *Dioscorea bulbifera*	(Adomėnienė and Venskutonis 2022; Uthirapathy 2021; Zhao et al. 2019)
Alkaloids	Diosbulbin B, ethyl iso‐allocholate, dumetorine, dioscorine, allantoin, dioscoretine, dihydrodioscorine	Antimicrobial,antihypertensive, anticancer, antiinflammatory, anti‐HIV	7.2–16 mg/100 g dry mass	(Obidiegwu et al. 2020; Padhan and Panda 2020; Wang et al. 2023; Zhao et al. 2019)
Flavonoids	Kaempferol‐3‐O‐rhamnoside, rutin, quercetol, kaempferol, catechin	Antimutagenic, antiinflammatory, antioxidant, anticarcinogenic, enzymatic regulation	0.62–0.85 mg/g dry weight in other *Dioscorea* species	(Padhan and Panda 2020; Panche et al. 2016; Zhao et al. 2019)
Phenolic compounds	Gentisic acid glycoside, 1,7‐bis‐(4‐hydroxyphenyl) hepta‐4,6‐dien‐3‐one, vanillic acid, syringic acid, protocatechuic acid, gallic acid, chlorogenic acid, 3‐methylphenol	Immunological control, antioxidants, antiinflammatory	297.3 mg/mL in *D. oppositifolia* ; 2.1–9.62 mg/g dry weight in various *Dioscorea* species	(Padhan and Panda 2020; Zhao et al. 2019)

## 6 Pharmacological Activities

**FIGURE 7:**
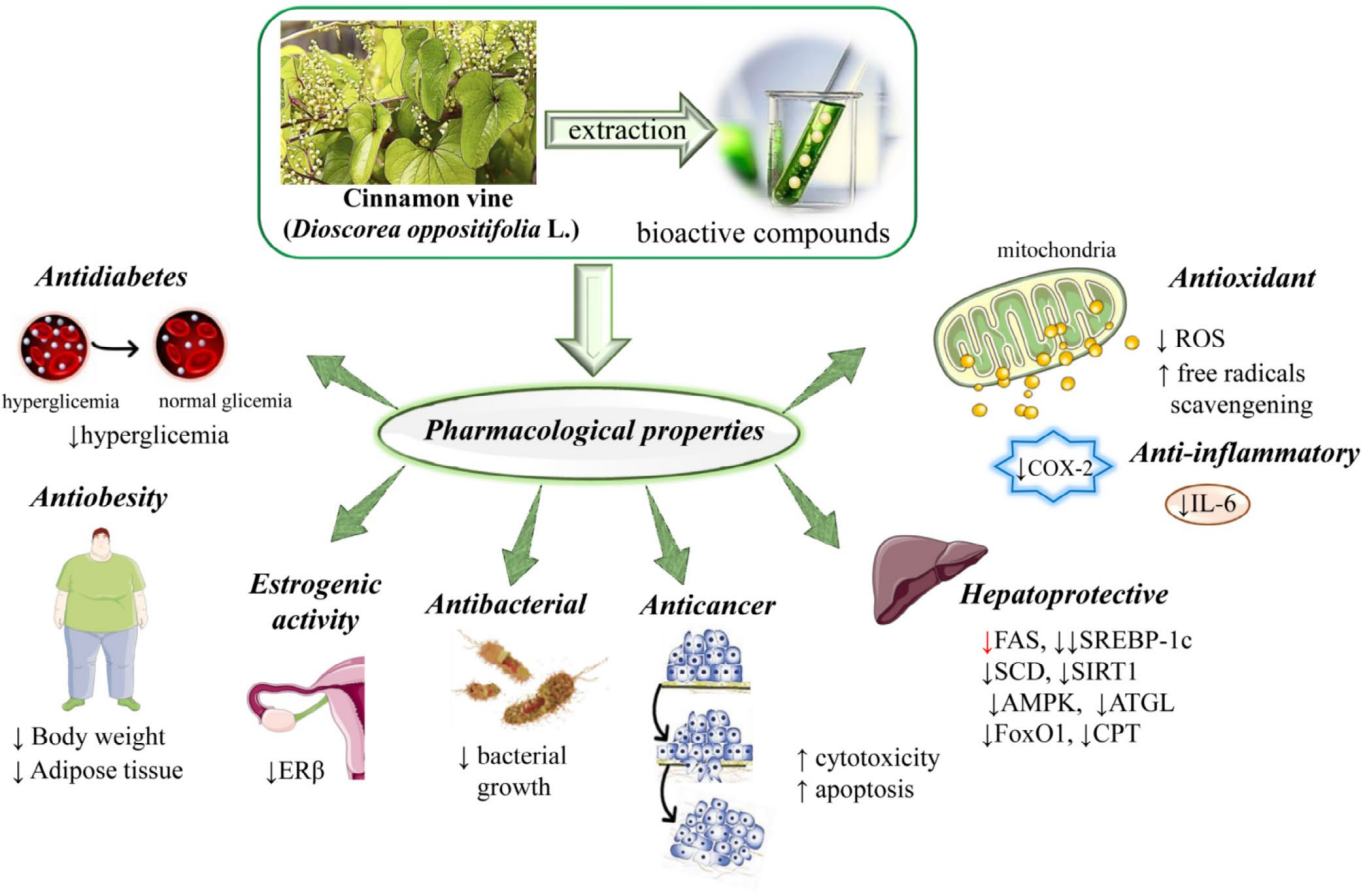
Pharmacological activities of *D. oppositifolia* . AMPK, AMP‐activated protein kinase; ATGL, adipose triglyceride lipase; COX‐2, cyclooxygenase‐2; CPT, carnitine palmitoyltransferase; ERβ, estrogen receptor beta; FAS, fatty acid synthase; FoxO1, forkhead box O1; IL‐6, interleukin 6; ROS, reactive oxygen species; SCD, stearoyl‐CoA desaturase; SIRT1, sirtuin 1; SREBP‐1c, sterol regulatory element‐binding protein 1c.

Different studies have revealed that the contents collected from various parts of *D. oppositifolia* can be used to cure a range of illnesses. It demonstrates anti‐inflammatory, anti‐carcinogenic, antiobesity, anti‐diabetic, antifertility, antioxidant, antimicrobial, and antifungal properties, illustrated in Figure 7 and Table 4.

### 6.1 Antioxidant

Antioxidants are substances that shield cells from oxidative damage and oxidation by scavenging oxygen radicals or hydroxy radicals (Roy et al. 2023). Phenols are important plant metabolites with hydroxyl groups and are the main contributors to antioxidant activity (Amin et al. 2022). Recent research on *D. oppositifolia* has demonstrated that the plant's tuber extract demonstrates free radical scavenging activity in all in vitro assay types in a dose‐dependent manner. *D. oppositifolia* free radical scavenging activity has been determined using DPPH, superoxide, hydroxyl, and ABTS in vitro free radical scavenging paradigms. As a result, the IC_50_ values for plant extract, standard ascorbic acid/trolox for DPPH, hydroxyl, superoxide, and ABTS scavenging activities were determined to be (21.47 μg/mL) and (18.26 μg/mL); (26.33 μg/mL) and (18.46 μg/mL); (31.59 μg/mL) and (72.08 μg/mL); (26.33 μg/mL) and (20.67 μg/mL). *D. oppositifolia* extract and ascorbic acid/Trolox concentration increases were also associated with a dose‐dependent increase in reductive capacity (Paulpriya and Mohan 2012). According to reports, *D. oppositifolia* extracts contain 0.51 g/100 g flavonoids and 0.56 g/100 g total phenols. Wang et al. (2020), reported protective and antioxidant impacts on the liver of rainbow trout, via using *D. oppositifolia* extract. A study by Padhan and Panda 2020, assessed the antioxidant activity of 9 distinct species of *Dioscorea* ( *D. bulbifera* , *D. pubera*, *D. alata* , *D. pentaphylla* , *D. oppositifolia* , *D. glabra* , *D. hispida* , *D. hamiltonii* and *D. wallichii* ) and revealed that the antioxidant activity ranges from 1.63% to 5.59%, having IC_50_ values of 77.9–1164, 101–1032, 27–1023, and 47–690 g/mL for DPPH, ABTS, superoxide, and nitric oxide scavenging activities, respectively (Padhan and Panda 2020).

### 6.2 Anti‐Inflammatory

The term anti‐inflammatory describes a treatment or substance's ability to lessen inflammation (Rahaman et al. 2023). Numerous processes contribute to inflammatory responses that involve the manufacture of thromboxanes, prostaglandins, leukotrienes, and prostacyclins by activating cyclooxygenases (COXs) and lipoxygenases (LOXs) (Yang et al. 2009). In a rat paw edema model caused by carrageenan, the methanolic extract of *D. oppositifolia* at 250 mg per kg body weight demonstrates greater potential against inflammation than the aqueous extract at 100 mg per kg body weight, with an anti‐inflammatory impact of 63.15% when tuber methanol was employed at a dosage of 250 mg per kg of body weight (Rani and Raju 2020). Zhao et al. found a correlation between the anti‐inflammatory impact of *D. oppositifolia* and a decrease in TNF‐α and IL‐6 expression levels. The treatment of *D. oppositifolia* lowers all of the inflammatory cytokine levels, and when compared to low groups, the high dosage of *D. oppositifolia* demonstrates superior effectiveness. Zhao et al. reported the anti‐inflammatory activity of two *D. oppositifolia* and *D. halmitonii* at the dose of 2 and 6 g/kg in which *D. oppositifolia* exhibits better inhibition than that of *D. halmitonii* (Zhao et al. 2019).

### 6.3 Anticancer

**FIGURE 8:**
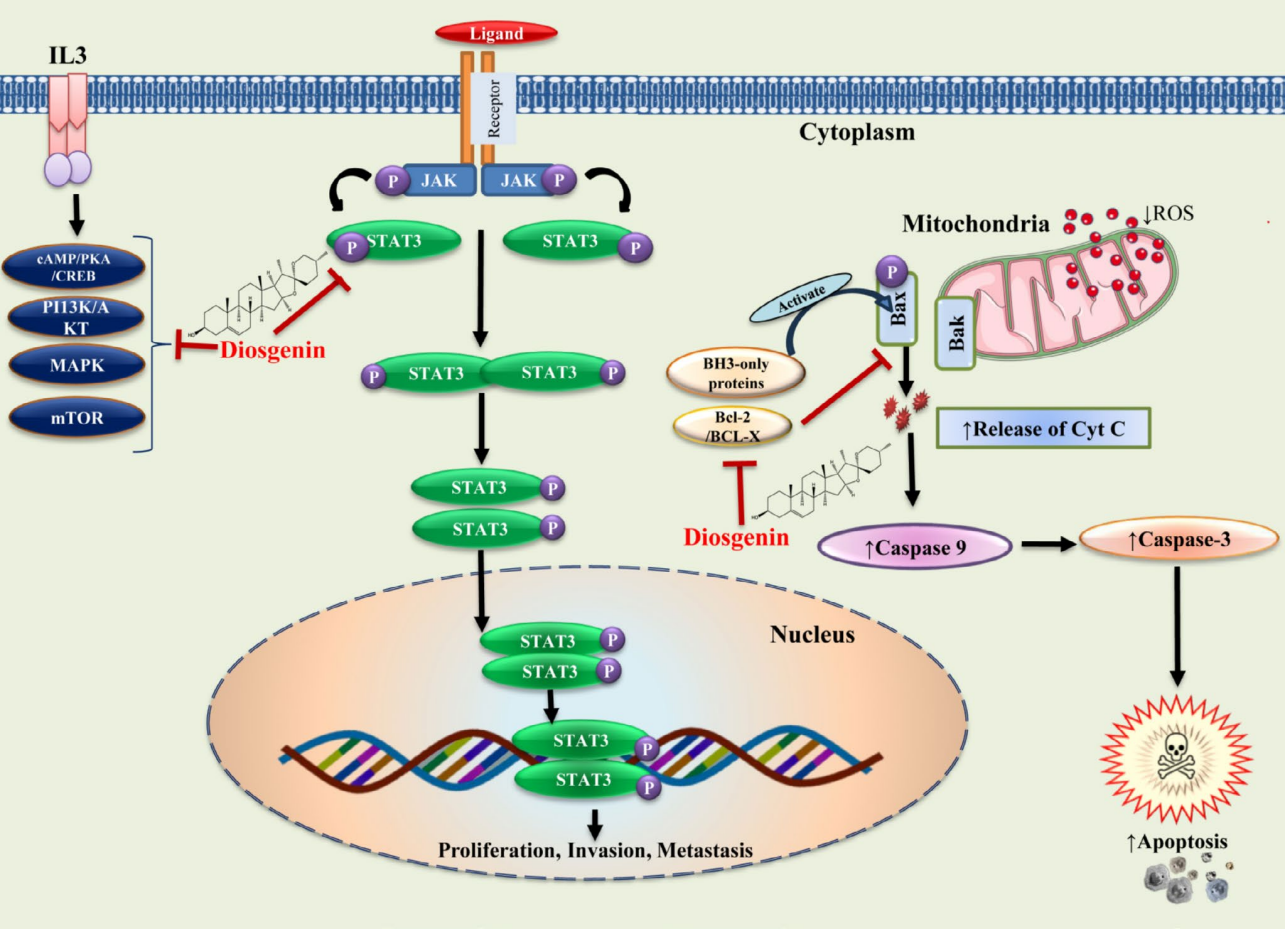
Diosgenin regulates cancer progression by modulating multiple pathways (i) P13K/Akt/mTOR pathway (ii) JAK/STAT pathway and (iii) via triggering apoptosis. This figure illustrates the impact of diosgenin, a bioactive compound found in *Dioscorea oppositifolia* (Cinnamon vine), on cell signaling pathways. At the cell surface, diosgenin inhibits the JAK–STAT pathway, reducing phosphorylation of STAT3 and preventing its action in the nucleus. Within the nucleus, inhibited STAT3 phosphorylation decreases transcription of genes involved in cell proliferation, invasion, and metastasis. In the mitochondria, diosgenin induces apoptosis by activating pro‐apoptotic proteins (Bax), inhibiting anti‐apoptotic proteins (Bcl‐2/Bcl‐X), leading to cytochrome c release, which activates caspase‐9 and caspase‐3, culminating in programmed cell death. ABTS, 2,2′‐azino‐bis(3‐ethylbenzothiazoline‐6‐sulfonic acid); AMPK, AMP‐activated protein kinase; ATGL, Adipose triglyceride lipase; Bax, Bcl‐2‐associated X protein; Bcl‐2, B‐cell lymphoma 2; Bcl‐X, B‐cell lymphoma‐extra large; BH3, Bcl‐2 homology domain 3; cAMP/PKA/CREB, Cyclic Adenosine Monophosphate/Protein Kinase A/CAMP Response Element‐Binding Protein; Caspase‐3, Cysteine‐aspartic proteases 3; Caspase‐9, Cysteine‐aspartic proteases 9; Cyt C, Cytochrome c; IL3, Interleukin 3; JAK, Janus Kinase; MAPK, Mitogen‐Activated Protein Kinase; mTOR, Mechanistic Target Of Rapamycin; PI3K/AKT, Phosphoinositide 3‐Kinases/Protein Kinase B; ROS, Reactive Oxygen Species; STAT3, Signal Transducer and Activator of Transcription 3.

Cancer is characterized by the uncontrolled growth and spread of abnormal cells, which can originate in one part of the body and then invade or spread to other areas (Buga et al. 2019). This complexity underscores the importance of understanding tumor heterogeneity, as evidenced in recent histochemical and immunohistochemical studies of colorectal cancer, to develop more targeted and effective therapeutic strategies (Zlatian et al. 2015). Natural bioactive substances found in plants are increasingly preferred alternatives to synthetic drugs and chemotherapy for the treatment of cancer, as they have fewer adverse effects that are frequently unbearable for the majority of cancer patients. Zeng et al. (2021) reported that non‐small cell lung cancer in vitro can be inhibited by a new Diphenylethane (D1) (2′,3,5‐Trihydroxy‐4‐Methoxybibenzyl) compound derived from leaves and stems of *D. oppositifolia* via the ERβ‐STAT3 Pathway as illustrated in Figure 8. D1 was found to lower the A549 cells' motility, viability, energy metabolism, and triggers apoptosis. Mechanistic research studies have revealed that D1 lowers the nuclear localization of STAT3 and suppresses STAT3 target gene production that includes Bcl‐xL, Mcl‐1, and MMP‐2, which contribute to the motility and survival of cells. Furthermore, their findings revealed that D1 possessed estrogenic actions that were Erβ mediated, and antagonizing Erβ reduced D1's cytotoxic effect on A549 cells. The suppression of nuclear translocation of STAT3 did not affect the interaction of Erβ and D1, while the nuclear translocation of STAT3 upregulates after antagonizing ERβ, thereby indicating that STAT3 was ERβ downstream signaling molecule (Zeng et al. 2021). Additionally, diosgenin is also studied for its potential to have therapeutic/chemopreventive effects against cancer in various organs, demonstrating the significant relevance of this as a possible antitumor agent (Ren et al. 2023). Diosgenin has been demonstrated to trigger numerous pathways of carcinogenesis that include proliferation, programmed cell death, invasion, tumor‐induced immune suppression, and angiogenesis in numerous tumorous cells (Raju and Rao 2012). Diosgenin has been evaluated for its ability to combat cancer in many tumor cell lines and has been discovered that its anticancer activity relies on the concentration and cell type (Bhardwaj et al. 2021). Thus, diosgenin inhibits cancer cell proliferation of Squamous carcinoma (A431, Hep2, and RPMI 2650 cells), Prostate cancer (PC‐3 and DU‐145 cells), Gastric cancer (BGC‐823 cells), Colon carcinoma (HCT‐116 and HT‐29 cells), Breast cancer (MCF‐7), Erythroleukemia (HEL cells), Lung cancer (A549 cells), Hepatocellular carcinoma (HepG2 and HCC cells) and Human chronic myeloid leukemia (CML) (K562 cells) (Mustafa et al. 2018). Also, diosgenin is shown to drastically reduce the development in Sarcoma‐180 cells in vivo while increasing the capacity of macrophages to phagocytose in vitro, and substantially enhance TNF‐α and NO secretion in the macrophages, thus indicating that diosgenin has the potential to upregulate the cellular Immune responses that are specific (He et al. 2012). Diosgenin halts the STAT3 signaling pathway, which results in the reduction of cellular differentiation, chemosensitization, and causes cell cycle halt at the G1 phase, triggers PARP cleavage, and programmed cell death (Li et al. 2010). Diosgenin suppresses the P13k/Akt/mTOR signaling pathway and causes preferentially cytotoxic effects in tumor cells (Bhardwaj et al. 2021).

### 6.4 Estrogenic Activity

Many natural compounds have been found to have estrogenic activity, in which hormone action is combined inadvertently or on purpose with unintended effects on physiology/endocrinology, brain function, developmental processes, and behavioral changes (Kiyama 2019). All *D. oppositifolia* compounds exhibit estrogenic activity and the compound was determined using a proliferation assay on MCF‐7 cell lines. Compounds such as Batatasin V, 3,3′,5‐trihydroxybibenzyl, Batatasin I, 2′,4‐dihydroxy‐2,5‐dimethyloxybibenzyl, and 6,7‐dihydroxy‐2,4‐dimethoxyphenanthrene boost ER expression and proliferation in MCF‐7 cell lines. Moreover, in MCF‐7 cell lines, the ERβ expression was entirely eradicated via the suppression of ERβ indicating that the effect of these compounds on proliferation was mediated through ERβ (Ren et al. 2019). Ren et al. have discovered two new nor sesquiterpenoids as Dioscopposin A and Dioscopposin B, along with twenty‐one other identified compounds, from the *D. oppositifolia* leaves and stems and assessed their estrogenic action on MCF‐7 cell lines, and found that 8 of the identified compounds reduced cell proliferation (Ren et al. 2021). Menopause is brought on by a reduction in the amount of estrogen secreted by ovaries and has a detrimental impact on the mind and body that includes hot flashes, osteoporosis, skin aging, and cardiovascular problems (Burger et al. 2007; Uthirapathy 2021). *Dioscorea* species have reportedly been shown to offer medical benefits that can help with menopausal symptoms in addition to serving as nutritional supplements. A Chinese anti‐menopausal medicine formulation containing *D. oppositifolia* rhizomes has been shown to modulate follicle‐stimulating hormone, luteinizing hormone, and estrogen levels in the blood, thereby reducing some negative impacts in postmenopausal women. It is persistent with the Lu et al. findings, who discovered *D. oppositifolia* to be effective in treating menopausal problems in Chinese medicine and reported that the bioactive proteins extracted from *D. oppositifolia* that stimulate estrogen boost estradiol synthesis and raise the expression of the Erβ, steroidogenic acute regulatory protein, and aromatase protein (Lu et al. 2016). Proteins extracted from *D. oppositifolia* can enhance the translational levels of ERβ, perhaps lowering the risk of developing ovarian malignancy (Obidiegwu et al. 2020).

### 6.5 Antibacterial and Antifungal

Human medicine has advanced significantly over time, but diseases brought on by viruses, parasites, bacteria, and fungi still pose a challenge (Taheri et al. 2021; Ungureanu et al. 2017). The antimicrobial potential of *D. oppositifolia* has been researched and reported as plant‐sourced antibiotic research has increased (Begum and Anbazhakan 2013). The solvent extract (methanol, ethyl alcohol, aqueous extract) of *D. oppositifolia* has been evaluated at various concentration levels (0.25, 0.50 and 1 mg/mL) in contrast to microorganisms that affect humans (Gram‐negative, Gram‐positive, and fungal strains) for their antibacterial a efficacy. The methanolic extract compared to the positive control significantly exhibited a strong inhibitory impact on *S. enterica* of 20 mm. The ethyl acetate extract showed a maximum inhibitory zone (20 mm) against *C. albicans* and the lowest inhibitory zone (13 mm) against *M. luteus* . The ethyl and methanolic extracts had the greatest antibacterial efficacy, whereas the aqueous extract had the least activity against every tested microorganism, with an inhibitory zone ranging from 8 to 17 mm. At 1 mg/mL, the zones of inhibitory activity of solvent extracts were greater than those at 0.25 and 0.5 mg/mL. A concentration‐dependent performance was seen in the antibacterial activity of several solvent extracts (Rani and Raju 2020). Female and male tubers of *D. oppositifolia* were found to be effective against the specified fungal strains as well as *Klebsiella pneumoniae* bacterium (Paul et al. 2020).

### 6.6 Effects on Metabolic Diseases

#### 6.6.1 Antidiabetic

Diabetes, a chronic condition characterized by high blood sugar levels, is often managed with medication and lifestyle changes (“Global, regional, and national burden of diabetes from 1990 to 2021, with projections of prevalence to 2050: a systematic analysis for the Global Burden of Disease Study 2021,” 2023). Bioactive natural compounds have shown promising antidiabetic effects, aiding in glucose regulation and improving insulin sensitivity, thus potentially serving as adjunct therapies for diabetes management (Quispe et al. 2022). *D. oppositifolia* contains diosgenin and has long been demonstrated to actively contribute to the management of diabetes in traditional medications. Diosgenin substantially lowers plasma glucose in diabetic rats induced via streptozotocin compared with other diabetic controls. Studies on the accumulation of lipids in type 2 diabetic rats in 3 T3‐L1 preadipocytes have demonstrated that diosgenin (at quantities varying between 0.1–10 μmol·L^−1^) enhances both adipocyte differentiation and PPARγ (peroxisome proliferative‐activated receptor gamma) expression, which result in a diosgenin hypolipidemic effect. Chronic inflammation in adipose tissue has been linked to type 2 diabetes and insulin resistance has been demonstrated to relate to obesity (Mustafa et al. 2018).

#### 6.6.2 Antiobesity

An accumulation of too much body fat as a result of an energy imbalance is one of the symptoms of the disorder known as obesity. Being a major public health concern, obesity contributes to subsequent persistent conditions such as dyslipidemia, cardiovascular disorders, and type 2 diabetes (Roh and Jung 2012). Jeong et al. (2016) reported that *D. oppositifolia* extract had an anti‐obesity effect on obese mice induced via diet. In their study, female mice were given a diet rich in fat for 3 weeks while also receiving 100 mg per kg *D. oppositifolia* n‐butanol extract as well as 15 mg per kg orlistat as a positive regulator. The researchers discovered that female mice who consumed *D. oppositifolia* n‐butanol extract had a consequent reduction in overall body weight and parietal adipose tissue weight, along with a decline in the levels of triglycerides, total cholesterol levels and LDL in blood serum The impact of n‐butanol extract of *D. oppositifolia* is intervened via the reduction of ingesting effectiveness and absorption of dietary fats. *D. oppositifolia* aqueous and methanolic extracts aid in the control of adipocyte differentiation via reducing the mRNA expression of FAS (fatty acid synthase), C/EBPα (CCAAT/enhancer‐binding protein alpha), SREBP‐F1 (sterol regulatory element‐binding protein F1), PPARγ (peroxisome proliferator‐activated receptor), and elevating the lipolytic CPT‐1 (carnitine palmitoyl transferase‐1) gene (Patil et al. 2022).

### 6.7 Effects on Gastrointestinal and Hepatic Disorders

#### 6.7.1 Gastroprotective

Peptic ulcers, manifesting as sores on the stomach's inner lining (gastric ulcers) or in the duodenum (duodenal ulcers), pose significant gastrointestinal challenges (Byeon et al. 2018; Dhasan et al. 2010). The anti‐ulcer efficacy of *D. oppositifolia* methanolic extract was tested in rats with ethanol and indomethacin‐induced ulcers. The extract‐treated groups showed significant inhibition of the ulcer index in contrast to the control group. Thus, as a result, *D. oppositifolia* exhibits substantial anti‐ulcer activity (Kumar et al. 2012). Furthermore, *D. oppositifolia* has anti‐ulcer properties that affect adult Wistar rats (Singh 2022).

#### 6.7.2 Hepatoprotective

The liver plays a pivotal role in detoxification, metabolism, and synthesis of critical proteins, exposing it to various damaging agents, including toxins and pathogens (Cortesi et al. 2023). In response, natural compounds from plants have garnered attention for their hepatoprotective properties, leveraging antioxidants and bioactive molecules to counteract liver damage. Despite these defenses, the liver remains vulnerable to cancer, particularly hepatocellular carcinoma, emphasizing the importance of integrated strategies that combine liver protection with targeted approaches to mitigate cancer risk (Jain et al. 2021). Dioscin, Diosgenin, polyphenols, and other compounds found in the *Dioscorea* extract have remarkable curative and preventative effects on Non‐alcoholic fatty liver disease. In both in vivo and in vitro studies, dioscin substantially downregulates hepatic lipid deposits and enhances hepatic biochemical indices and serum levels (Wang et al. 2023). Dioscin lowers the expression of FAS, SREBP‐1c, SCD, and SIRT1 (Silent information regulator of transcription factor 1), AMPK (AMP‐activated protein kinase), ATGL (Adipose Triglyceride Lipase), FoxO1 (Forkhead Box O 1) and CPT (Carnitine palmitoyltransferase), which are the downstream proteins whose expression levels are altered via dioscin (Yao et al. 2018). While diosgenin, in order to decrease high glucose‐induced TG buildup, suppresses LXRα and stimulates the AMPK pathway to prevent the buildup of hepatic lipids in both in vivo and in vitro studies (Parama et al. 2020).

**TABLE 4:** Pharmacological activities of *D. oppositifolia* .

Pharmacological activities	Tested model/system	Tested extract type	Effect/outcome	Mechanism	IC_50_ concentration/dose	References
Antioxidant	In vitro assays	Tuber	↑ Scavenging activity	Free radical scavenging via DPPH, superoxide, hydroxyl, and ABTS assays	DPPH: 21.47 μg/mL; hydroxyl: 26.33 μg/mL; superoxide: 31.59 μg/mL; ABTS: 26.33 μg/mL	(Amin et al. 2022; Lubag et al. 2008)
Anti‐inflammatory	In vivo, rat paw edema	Methanolic	↓ Inflammation	↓ TNF‐α, ↓IL‐6, dose‐dependent efficacy	Dose = 250 mg/kg (63.15% efficacy)	(Rani and Raju 2020; Zhao et al. 2019)
Anticancer	In vitro, A549 cells	Dichloromethane extract	↓ Cell motility/viability, ↑ apoptosis	↓ ERβ‐STAT3, ↓ STAT3 nuclear localization	IC_50_ = 38.9 μM and 29.2 μM	(Zeng et al. 2021)
Estrogenic	In vitro, MCF‐7 cells	Dichloromethane extract	↑ ER expression, ↓ cell proliferation	↓ ERβ	Dose = 10 μM	(Ren et al. 2021; Ren et al. 2019)
Antimicrobial	*Micrococcus luteus* MTTC 2470, *Klebsiella pneumoniae* MTCC 7028, *Pseudomonas aeruginosa* MTCC 7296, *Salmonella enterica* MTCC 98, *Fusarium oxysporum* MTCC1272, and *Candida albicans* MTTC 854	Methanolic, ethyl acetate, aqueous	Variable inhibitory zones	Concentration‐dependent efficacy	Inhibitory zones: 8–20 mm (0.25, 0.50 and 1 mg/mL)	(Paul et al. 2020; Rani and Raju 2020)
Anti‐diabetic	In vivo, diabetic rats (STZ‐induced)	Diosgenin compound	↓ Plasma glucose, ↑ insulin sensitivity	↑ Adipocyte differentiation, ↑ PPARγ	0.1–10 μmol L^−1^	(Mustafa et al. 2018)
Anti‐obesity	In vivo, obese mice (diet‐induced)	n‐butanol	↓ Body weight, ↓ adipose tissue	Reduces dietary fat absorption, modulates gene expression related to fat metabolism	Dose = 100 mg/kg	(Jeong et al. 2016; Patil et al. 2022)
Gastroprotective	In vivo, mice	Aquous, ethanolic	↓ Plasma, TNFα level	Significant alteration in the gastric epithelium, reflecting hemorrhagic necrosis and collapse of the gastric mucosa with epithelial cell loss, contributed to preserving the structure of gastric wall	Dose = 100 or 200 mg/kg body weigh	(Byeon et al. 2018; Kumar et al. 2012; Singh 2022)
Hepatoprotective	In vitro (AML‐12 cells), in vivo (male Wistar rats/mice)	Dioscin dissolved with 0. 1% dimethylsulfoxide (DMSO) for the in vitro experiments and with 0.5% carboxymethylcellulose sodium (CMC‐Na) solution for the in vivo tests	↓ Hepatic lipid deposits, ↑ liver function	↓ FAS, ↓ SREBP‐1c, ↓ AMPK	150, 300, 600 ng/mL for AML‐12 cells, high‐dose dioscin group (80 mg/kg for mice and 60 mg/kg for rats), low‐dose dioscin group (20 mg/kg for mice and 15 mg/kg for rats)	(Parama et al. 2020; Wang et al. 2023; Yao et al. 2018)

Abbreviations: ↑, increase; ↓, decrease; ABTS, 2,2′‐azino‐bis(3‐ethylbenzothiazoline‐6‐sulfonic acid); AMPK, AMP‐activated protein kinase; DPPH, 2,2‐diphenyl‐1‐picrylhydrazyl; ERβ, estrogen receptor beta; FAS, fatty acid synthase; IL‐6, interleukin 6; NAFLD, non‐alcoholic fatty liver disease; PPARγ, peroxisome proliferator‐activated receptor gamma; SREBP‐1c, sterol regulatory element‐binding protein 1c; STZ, streptozotocin; TNF‐α, tumor necrosis factor alpha.

## 7 Limitations

The current review highlights the diverse pharmacological activities of *D. oppositifolia* , including antioxidant, anti‐inflammatory, anti‐obesity, hepatoprotective, anti‐cancer, estrogenic, antimicrobial, anti‐diabetic, and anti‐ulcer effects, but several limitations and clinical gaps need to be investigated:

- There is a significant lack of clinical trials to substantiate the therapeutic efficacy of *D. oppositifolia* in humans and rigorous clinical studies are essential to validate the traditional and experimental therapeutic claims associated with this plant.
- The variability in the chemical composition of *D. oppositifolia* extracts due to differences in geographical origin, harvesting time, and extraction methods poses challenges in standardizing the extracts. This variability directly impacts the reproducibility of pharmacological findings and the establishment of precise dosages for therapeutic applications.
- Comprehensive toxicological evaluations are necessary to ascertain the safety profile of *D. oppositifolia* extracts and compounds. Acute and chronic toxicity studies, along with assessments of potential mutagenic and carcinogenic effects, are important to ensure the safe use of these plant‐derived compounds in clinical settings.
- Although several studies have elucidated the potential mechanisms of action of various bioactive compounds found in *D. oppositifolia* , a deeper understanding of these mechanisms at the molecular level is needed. Such insights are vital for the development of targeted therapies and for elucidating potential interactions with conventional medications.
- Limited information is available on the bioavailability, pharmacokinetics, and metabolism of the active compounds of *D. oppositifolia* . Research in this area is essential for optimizing the delivery and efficacy of these compounds in clinical applications.
- Traditional uses of *D. oppositifolia* often involve whole plant extracts, which contain a complex mixture of bioactive compounds; understanding the synergistic or antagonistic interactions among these compounds can provide insights into the holistic efficacy of the plant and contribute to the development of more effective therapeutic agents.

## 8 Safety and Toxicity

The traditional medical system utilizes natural remedies to treat a wide range of ailments safely and effectively, as long as they are used as directed and are under the supervision of a skilled medical professional (Kumari et al. 2022). Wistar albino rats were used in acute toxicity experiments with methanolic and aqueous *D. oppositifolia* tubers extract in a single oral dose that failed to exhibit any sign of substantial toxicity when evaluated for the first 4 h, and then everyday observation for the next 14 days revealed no mortality. Furthermore, no symptoms or indications of changed appetite or body weight, restlessness, respiratory distress, psychomotor activity, diarrhea, convulsions, or coma were present. According to the OECD guidelines 425 (procedure for evaluating Acute Oral Toxicity), it was demonstrated to be safe up to a dose of 5000 mg per kg body weight. As a result, 1/5th and 1/10th doses of 5000 mg/kg (LD50) of 1000 mg and 500 mg per kg body weight can be utilized as the safest doses in the experimental studies (Rani and Raju 2020).

## 9 Conclusion

The current review provides numerous details about the morphological, ethnobotanical, phytochemical, and pharmacological characteristics of *D. oppositifolia*. Several types of active phytoconstituents are present in *D. oppositifolia* , a significant plant that contains an array of bioactive molecules, such as flavonoids, saponins, tannins, and polyphenols, which have been demonstrated to have beneficial effects on several diseases and disorders, including anti‐carcinogenic, anti‐ulcer, anti‐inflammatory, and anti‐microbial, which explains their potential significance to cure a variety of medical ailments. This plant possesses a lot of nutritional and medicinal potential; thus, it might function as a better option than synthetic medications to treat a variety of illnesses, but there is little knowledge about the complete mode of action of its bioactive compounds. Nowadays, foods with dietary potential and secondary metabolites are focused on because of dual benefits. Tubers are good sources for both dietary as well as secondary metabolites. Nutritional profiling of *D. oppositifolia* reveals that it possesses higher quantities of carbohydrates, fiber, starch, and sugar, making it a possible dietary source and possesses numerous secondary metabolites. It can be a very useful plant because of its nutritional as well as therapeutic uses. The current study aims to fill knowledge gaps and provide sources of information for researchers working in clinical studies and phytomedicine.

## Author Contributions

**Ruchika Kumari:** data curation (equal), investigation (equal), methodology (equal), writing – original draft (equal), writing – review and editing (equal). **Ankita Thakur:** data curation (equal), investigation (equal), methodology (equal), writing – original draft (equal), writing – review and editing (equal). **Palak Thakur:** data curation (equal), investigation (equal), methodology (equal), writing – original draft (equal), writing – review and editing (equal). **Vipasha Sharma:** data curation (equal), investigation (equal), methodology (equal), writing – original draft (equal), writing – review and editing (equal). **Rohit Sharma:** data curation (equal), investigation (equal), methodology (equal), writing – original draft (equal), writing – review and editing (equal). **Sachin Upmanyu:** data curation (equal), investigation (equal), methodology (equal), writing – original draft (equal), writing – review and editing (equal). **Randeep Singh:** data curation (equal), investigation (equal), methodology (equal), writing – original draft (equal), writing – review and editing (equal). **Zainab M. Almarhoon:** data curation (equal), investigation (equal), methodology (equal), writing – original draft (equal), writing – review and editing (equal). **Daniela Calina:** data curation (equal), investigation (equal), methodology (equal), project administration (equal), supervision (equal), validation (equal), visualization (equal), writing – original draft (equal), writing – review and editing (equal). **Javad Sharifi‐Rad:** conceptualization (equal), data curation (equal), formal analysis (equal), investigation (equal), methodology (equal), project administration (equal), supervision (equal), validation (equal), visualization (equal), writing – original draft (equal), writing – review and editing (equal). **Ashun Chaudhary:** data curation (equal), formal analysis (equal), investigation (equal), methodology (equal), project administration (equal), supervision (equal), validation (equal), visualization (equal), writing – original draft (equal), writing – review and editing (equal).

## Conflicts of Interest

The authors declare no conflicts of interest.

## Data Availability

The authors have nothing to report.
